# Excretory/Secretory-Products of *Echinococcus multilocularis* Larvae Induce Apoptosis and Tolerogenic Properties in Dendritic Cells *In Vitro*

**DOI:** 10.1371/journal.pntd.0001516

**Published:** 2012-02-21

**Authors:** Justin Komguep Nono, Katrien Pletinckx, Manfred B. Lutz, Klaus Brehm

**Affiliations:** 1 University of Würzburg, Institute of Hygiene and Microbiology, Würzburg, Germany; 2 University of Würzburg, Institute of Virology and Immunobiology, Würzburg, Germany; University of Edinburgh, United Kingdom

## Abstract

**Background:**

Alveolar echinococcosis, caused by *Echinococcus multilocularis* larvae, is a chronic disease associated with considerable modulation of the host immune response. Dendritic cells (DC) are key effectors in shaping the immune response and among the first cells encountered by the parasite during an infection. Although it is assumed that *E.multilocularis*, by excretory/secretory (E/S)-products, specifically affects DC to deviate immune responses, little information is available on the molecular nature of respective E/S-products and their mode of action.

**Methodology/Principal Findings:**

We established cultivation systems for exposing DC to live material from early (oncosphere), chronic (metacestode) and late (protoscolex) infectious stages. When co-incubated with *Echinococcus* primary cells, representing the invading oncosphere, or metacestode vesicles, a significant proportion of DC underwent apoptosis and the surviving DC failed to mature. In contrast, DC exposed to protoscoleces upregulated maturation markers and did not undergo apoptosis. After pre-incubation with primary cells and metacestode vesicles, DC showed a strongly impaired ability to be activated by the TLR ligand LPS, which was not observed in DC pre-treated with protoscolex E/S-products. While none of the larvae induced the secretion of pro-inflammatory IL-12p70, the production of immunosuppressive IL-10 was elevated in response to primary cell E/S-products. Finally, upon incubation with DC and naïve T-cells, E/S-products from metacestode vesicles led to a significant expansion of Foxp3+ T cells *in vitro*.

**Conclusions:**

This is the first report on the induction of apoptosis in DC by cestode E/S-products. Our data indicate that the early infective stage of *E. multilocularis* is a strong inducer of tolerance in DC, which is most probably important for generating an immunosuppressive environment at an infection phase in which the parasite is highly vulnerable to host attacks. The induction of CD4+CD25+Foxp3+ T cells through metacestode E/S-products suggests that these cells fulfill an important role for parasite persistence during chronic echinococcosis.

## Author Summary

Parasitic helminths are inducers of chronic diseases and have evolved mechanisms to suppress the host immune response. Mostly from studies on roundworms, a picture is currently emerging that helminths secrete factors (E/S-products) that directly act on sentinels of the immune system, dendritic cells, in order to achieve an expansion of immunosuppressive, regulatory T cells (T-reg). Parasitic helminths are currently also intensely studied as therapeutic agents against autoimmune diseases and allergies, which is directly linked to their immunosuppressive activities. The immunomodulatory products of parasitic helminths are therefore of high interest for understanding immunopathology during infections and for the treatment of allergies. The present work was conducted on larvae of the tapeworm *E. multilocularis*, which grow like a tumor into surrounding host tissue and thus cause the lethal disease alveolar echinococcosis. The authors found that E/S-products from early infective larvae are strong inducers of tolerogenic DC *in vitro* and show that E/S-products of larvae of the chronic stage lead to an *in vitro* expansion of Foxp3+ T cells, suggesting that both the expansion of these T cells and poorly responsive DC are important for the establishment and persistence of *E. multilocularis* larvae within the host.

## Introduction

The metacestode larval stage of the fox-tapeworm *E. multilocularis* is the causative agent of alveolar echinococcosis, one of the most dangerous zoonoses world-wide [1]. Apart from the strobilar adult stage that resides within the intestine of the definitive host (e.g. foxes, dogs), the life cycle of this cestode comprises three larval stages that are involved in the infection of the intermediate host (small rodents and, occasionally, humans). An infection of the intermediate host is initiated by the oral uptake of ‘infectious eggs’ that contain the first larval stage, the oncosphere. Upon activation within stomach and intestine, the oncosphere hatches, penetrates the intestinal wall, and gains access to the host's viscera. Almost exclusively within the intermediate host's liver, the oncosphere then undergoes a metamorphosis towards the metacestodes which is driven by totipotent parasite stem cells (germinal cells; neoblasts) that were carried into the host through the oncosphere. As a result of the oncosphere - metacestode metamorphosis, fully mature, cyst-like metacestode vesicles are formed that grow infiltratively, like a malignant tumor, into the surrounding host tissue and that consist of an inner, cellular ‘germinal layer’(GL) and an outer, glycan-rich and acellular ‘laminated layer’ (LL) [2]. At least in experimentally infected mice, the formation of the LL cannot be observed earlier than 2–3 weeks upon initial infection [3], [4], [5], [6]. Evidence has been obtained that the LL is one of the parasite's key structures for protection against the host immune system in the later phase of the infection [7]. Approximately 2 months after the infection of mice, ‘brood-capsules’ are formed from stem cells of the GL that later give rise to the third larval stage, the protoscolex, which is passed on to the definitive host [2]. The *E. multilocularis* infection process can thus be separated into 3 phases. The first phase starts with the oncosphere and culminates, after 2–3 weeks, in the formation of mature metacestode vesicles which, in the second phase, grow infiltratively into the host tissue. During the third stage, protoscoleces are formed in natural intermediate hosts, but only rarely in human infections [2].

Cellular effector mechanisms are considered to be the key defense against metacestode growth and dissemination in mice and humans since mouse strains that cannot develop cellular immune responses are highly susceptible to AE, whereas strains defective in humoral immunity can control parasite growth to a certain level [8]. Furthermore, in humans co-infected with *E. multilocularis* and the human immunodeficiency virus (HIV), very fast and unlimited parasite proliferation occurs [9], [10], whereas promotion of cellular immunocompetence has a beneficial effect on the outcome of the disease [8]. A significant number of studies on both humans and mice indicated that T helper 1 (Th1)-dominated immune responses, characterized by the release of interferon-γ (IFN-γ), after priming by DC that secrete interleukin (IL)-12, are effective in eliminating the parasite at an early stage, whereas a Th2 cytokine profile (IL-4, IL-5) and the release of immunosuppressive IL-10 and TGF-β is generally associated with susceptibility to the parasite and progressive disease [8]. Although it became clear from these studies that the parasite, most probably by E/S-products, actively influences the host immune response (e.g. gradually driving it into the Th2 branch), little is currently known on the molecular and cellular basis of *E. multilocularis* induced immune suppression, particularly for the early stages of the infection.

DC are professional antigen presenting cells that represent the link between the innate and the adaptive immune system and are crucially involved in the induction of Th1-, Th2- or Th17-dominated immune responses [11], [12]. Upon pathogen recognition, DC take up antigens and undergo maturation, as can be assessed by the up-regulation of surface markers such as the major histocompatibility complex II (MHC II) and co-stimulatory molecules such as CD86 and CD80 [11]–[13]. After migration to the T cell area of lymph nodes, DC interact with naïve T cells to promote adaptive immune responses towards the Th1-, Th2-, Th17-branches, depending on the pathogen pattern they have adopted [13]. However, DC are also targets of parasites to establish immune evasion, e.g. by induction of regulatory T cells (T-reg), which counteract T helper cell activities [11], [12], [14]. A potentially important role of DC in immunosuppressive mechanisms during AE has indeed been established in a recent *in vivo* study on secondary (intraperitoneal) AE in mice [15]. In this work, Mejri *et al.* demonstrated that peritoneal DC from chronically infected mice, representing the late stage of AE, express higher levels of TGF-β mRNA, lower levels of IL-10 and IL-12 mRNA, and display down-regulation of maturation-associated surface markers, when compared to DC from non-infected mice [15]. Furthermore, DC from intraperitoneally infected mice specifically modulated CD4^+^ and CD8^+^ T cell responses suggesting a role for immunosuppressive T-reg during chronic AE [15]. DC are also among the first cells encountered by parasites during an infection [11], [13] and may have a critical role in the Th1 to Th2 shift reported for the intermediate host during the chronic phase of AE [8], [10]. In line with this hypothesis are recent data demonstrating that immature human DC fail to mature in the presence of crude, non-fractionated *E. multilocularis* antigen [16]. Moreover, during infection of the intermediate host, migration of parasitic larvae from the intestinal entry site to the liver and late metastasis to other organs (lung, brain) [17] strongly suggest that these larvae encounter DC *in vivo*. However and in spite of the general importance of DC in cellular host-helminth interaction mechanisms [11], [12], [18], only few investigations have so far been carried out towards an identification and characterization of immunomodulatory molecules that are released by *Echinococcus* larvae and their influence on DC function. Apart from the above mentioned study concerning the influence of crude *E. multilocularis* antigen on human DC [16], there are merely reports on the activity of crude hydatid (vesicle) fluid or selected hydatid fluid protein compounds of the related tapeworm *E. granulosus* on DC maturation [19], [20].

Due to the limited availability of respective parasite material (oncospheres), no *in vitro* studies have so far been carried out concerning the interaction of host immune cells with early infective parasite stages. Our current picture concerning the effects of *Echincoccus* E/S-products on host cells thus mostly derives from studies in which easier accessible protoscoleces had been employed [21]–[27], with the considerable drawback that this stage is formed very late during an infection of the intermediate host (if at all), and that in intact metacestode vesicles, protoscoleces do not have direct contact to host tissue and cells. Notably, we have recently introduced a primary germinal cell cultivation system by which the early developmental phase within the intermediate host can be re-constituted *in vitro* [28]. In this system, isolated *E. multilocularis* primary cells proliferate and form cellular aggregates that give rise to mature metacestode vesicles (including LL) in a manner that closely mimics the natural oncosphere - metacestode metamorphosis process [28]. Even concerning gene and protein expression patterns, this system closely reflects early parasite development within the intermediate host, and parasite antigens originally described to be expressed specifically in the oncosphere are readily detectable in the regenerating parasite cell aggregates [29], [30]. Although it became clear from previous studies that *E. multilocularis* through its larval E/S-products tightly down regulates accessory cell functions of macrophages [31] little is currently known about the effect on DC. In the present study, we used our primary germinal cell cultivation system [28] to investigate the influence of E/S-products from primary cells (characteristic of the early phase of the infection) on DC and compared it with the effects of E/S-products of mature metacestode vesicles, characteristic for the chronic phase, and protoscoleces which, in intact parasite material, do not have direct contact with host immune cells.

## Materials and Methods

### Ethics statement

All experiments were carried out in accordance with European and German regulations on the protection of animals (*Tierschutzgesetz*). Ethical approval of the study was obtained from the local ethics committee of the government of Lower Franconia (*Regierung von Unterfranken*; 621-2531.01-2/05 and 55.2-2531.01-73/07).

### Isolation and cultivation of *E. multilocularis* larvae

All experiments were performed with the natural *E. multilocularis* isolate JAVA [32] which was propagated in Mongolian jirds (*Meriones unguiculatus*) as described [33]. Isolation of metacestode tissue and axenic cultivation of metacestode vesicles was performed essentially as described previously by Spiliotis and Brehm [33]. For the isolation of protoscoleces, parasite tissue was isolated from infected jirds and homogenized as described [34]. The homogenate was subsequently filtered through a nylon mesh of 150 µm pore size, thus separating protoscoleces from larger pieces of metacestode tissue. The flow through was subsequently filtered through a nylon mesh of 30 µm pore size, separating protoscoleces from single cells and small cell clumps. Protoscoleces were then washed off the nylon mesh with sterile PBS and separated from equally sized metacestode vesicles by microscope-aided, manual picking with a pipette tip prior to applying axenic cultivation conditions in order to eliminate eventual host remnants [33] For the isolation of primary cells, axenically cultivated metacestode vesicles were mechanically sheared and trypsin-digested essentially as previously described [28]. Primary cells were then directly cultivated in hepatocyte-conditioned medium supplemented with reducing agents under a nitrogen atmosphere.

### Qualitative assessment of *E multilocularis* larval material

After one week of cultivation under axenic conditions [33], [35], the different larval stages (primary cells, metacestode vesicles, protoscoleces) were analyzed for host cell contamination by organism-specific PCR. Chromosomal DNA was isolated from the larvae and from liver tissue of a non-infected jird using the DNeasy isolation kit (Qiagen). Part of the parasite specific gene *elp* (ezrin-radixin-moesin-like [36]) was amplified using the primers Em10-15 (5′-TCC TTA CCT TGC AGT TTT GT -3′) and Em10-16 (5′-TTG CTG GTA ATC AGT CGA TC-3′). As a control for host-DNA contamination, a previously described β-tubulin-encoding gene from *Meriones unguiculatus* was used [37], employing primers Tub12-UP and TUB12-ST as described [38].

### Quantitative assessment of *E multilocularis* larvae and normalization

*In vitro* cultivated material of all three larval stages was isolated and cell lysates were generated by first passing larval material repeatedly through a pipette tip, followed by one washing step in 1×PBS, and subsequent treatment with 50 µl of 2× STOP mix (2 ml 0.5 M Tris–HCl pH 6.8, 1.6 ml glycerol, 1.6 ml 20% SDS, 1.4 ml H_2_O, 0.4 ml 0.05% (w/v) bromphenol blue, 7 µl β-mercapto-ethanol per 100 µl) and boiling for 10 min at 100°C. Proteins were separated by SDS-PAGE and analyzed by Western blotting using an antibody directed against β-actin (Cell signalling technology®; No. 4967) of a wide variety of metazoan organisms. Images were subsequently analyzed for the relative expression of β-actin using the Image-J program (http://rsb.info.nih.gov/ij/) [39]. The relative expression transcribed as values of area under the curve (AUC) was used to normalize the β-actin content of each sample. In a first set of experiments, different amounts of in vitro cultivated primary cells, metacestode vesicles and protoscoleces were analyzed (Figure S1A) and the relative β-actin content of each sample was then used as a basis for normalization. Based on previous studies showing that, *in vivo*, oncosphere-derived stem cells develop into mature metacestode vesicles within 2–3 weeks upon infection [3]–[6], we first determined the amount of primary cells which, in our *in vitro* system, lead to the production of metacestode vesicles within the same time. This was the case when we used 1/6^th^ of the amount of primary cells that can be isolated from 40 ml of metacestode vesicles (Figure S1B). This amount was defined as 1 Unit and contained ∼600 µg of total protein (Figure S1B). We then carried out calculations for the remaining larval stages and found that 2000 protoscoleces as well as 4 metacestode vesicles (2 months of age) of a diameter of 5 mm after 1 week of axenic cultivation represented 1 Unit and also contained ∼600 µg of total protein (Figure S1B). The reliability of this quantitative approach was further assessed by comparing one half unit of parasite material from each stage (i.e. 1000 protoscoleces, 2 metacestode vesicles of a diameter of 5 mm and 1/12^th^ of the amount of primary cells that can be isolated from 40 ml metacestode vesicles) which, as shown in Figure S1C, also resulted in comparable β-actin content.

### Mice

C57BL/6 mice were purchased from Charles River/Wiga (Sulzfeld, Germany) and TCR transgenic OT2 B6 mice were a kind gift of Prof. F. Carbone (Melbourne, Australia). All mice were bred within the animal facility of the Institute of Virology and Immunobiology, University of Würzburg, under specific pathogen-free conditions. Female mice were used at the age of 6–14 weeks.

### Splenocyte isolation

Single cell suspensions were obtained from the spleen of C57BL/6 mice by mechanically squeezing the tissue with glass slides in cold PBS and filtered through a 70 µm nylon cell strainer. Red blood cells in the filtrate were lysed with 1,4% NH_4_Cl for 5 minutes at 37°C, and the splenocytes were washed in R10 medium, that is RPMI 1640 (GIBCO BRL) supplemented with penicillin (100 U/ml, Sigma, Deisenhofen, Germany), streptomycin (100 µg/ml, Sigma), L-glutamine (2 mM, Sigma), 2-mercaptoethanol (50 µM, Sigma and 10% heat-inactivated fetal calf serum (FCS, PAA Laboratories, Parsching, Austria). Cell counts were subsequently determined using the trypan blue (No. 26323, Biochrom, Berlin, Germany) exclusion test on a bright-lined Neubauer counting chamber.

### Generation of murine bone marrow-derived DC

DC were generated from the bone marrow (BM) precursors of C57BL/6 mice as previously described [40]. Briefly, BM precursor cells were cultured for 8 days with GM-CSF. At day 8, non-adherent DC (70–80% CD11c+ cells) were harvested and seeded at a density of 10^6^ cells/ml R10 culture medium.

### Stimulation of murine DC and splenocytes with E/S-products of *E. multilocularis* larvae

A comparable amount of axenically cultivated parasite material, normalized for β-actin content, from each of the three larval stages was used throughout the stimulation process. Tissue culture inserts (Greiner Bio-One) of 1 µm pore size with or without larvae were thoroughly washed in R10 medium to completely remove hepatocyte-conditioned medium, were then added to DC or splenocyte cultures, and kept at 37°C in the presence of 5% CO_2_ for different time points. For LPS stimulation experiments, inserts containing parasite material were removed after 24 h, DC were harvested and re-plated at an equal number of living cells (5×10^5^ cells/ml) in R10 culture medium with or without 0.1 µg/ml lipopolysaccharide (LPS; *E. coli* 0127:B8; Sigma Aldrich) for additional 48 h. Upon completion, DC or splenocyte viability was determined by trypan blue exclusion (No. 26323, Biochrom, Berlin, Germany) on a bright-lined Neubauer counting chamber. Flow cytometric assessment of DC surface staining was then performed using fluorochrome-conjugated antibodies (anti-mouse) against the surface lineage marker CD11c (CD11c-PE-Cy5.5, Caltag Laboratories), MHC II (eBiosciences) and CD86 (B7-2-FITC, eBiosciences or B7-2-PE, BD Pharmingen). Splenocytes were stained for CD19 (CD19-pecy5, BD Pharmingen) as a specific marker for B cells and an exclusion marker for T cells within lymphocytes. To monitor the level of DC apoptosis, annexin-V binding buffer (BD Pharmingen) and FITC-conjugated annexin-V ready-to-use solution (BD Pharmingen) were used coupled to 7-AAD staining solution (BD Pharmingen). To assess DC maturation, marker-specific antibodies (CD11c, MHC II and CD86) were applied and after 30 min incubation at 4–8°C in the dark, cells were washed twice with FACS buffer (3% FCS, 0.1% NaN_3_ in PBS) and fixed with 1% (v/v) formaldehyde in PBS. The staining procedure was identically conducted for CD 19 on splenocytes. In DC apoptosis assays, stimulated DC along with UV-irradiated DC (positive control for apoptosis) at a peak intensity of 9000 mW/cm^2^ at the filter surface and a peak emission of 313 nm (trans-illuminator), were directly resuspended in 50 µl of 1× annexin-V binding buffer. Next, 5 µl of 7-AAD and 2 µl of annexin-V–FITC were added to the tubes and incubated for 15 minutes at RT. Cells were then resuspended in 200 µl of 1× annexin-V binding buffer then acquired on a FACSCalibur™ (Beckton Dickinson) cytometer, equipped with CellQuest software. Results were further analyzed with FlowJo software (Tree Star, USA).

### Measurement of cytokine release by DC

After stimulation of DC, the production of IL-6, IL-10, and IL-12p70 was measured in supernatant using sandwich enzyme-linked immunosorbent assays (ELISA, OptEIA kits, BD Pharmingen) according to the manufacturer's instructions. The kits detection limits were of 39 pg/ml for IL-12p70 and 19 pg/ml for IL-10 and IL-6.

### Preparation of OT2 T cells

Spleens and lymph nodes from 6–14 weeks old OT2 mice were isolated, and the separated splenocytes and lymph nodes cells, obtained as described above (Splenocytes isolation), were resuspended in cold PBS. CD4+ cells were isolated using an EasyStep negative selection mouse CD4+ T cell enrichment kit (Stemcell Technologies). After separation, the purity of T-cell preparation was routinely higher than 90%, as determined by flow cytometry. CD4+ T cells were subsequently enriched for CD25− cells using Miltenyi Biotec's LD columns with a suitable MACS separator usually achieving 90–95% of purity.

### *In vitro* regulatory T cell conversion assay

DC were incubated with 3-fold higher numbers of OT2 CD4+CD25− T cells and 200 ng/ml of OVA protein (Sigma, grade V) supplemented or not with parasite larvae E/S-products (supernatant of equal amounts of larvae kept in medium for 7 days). After 5 days of co-culture, the cells were harvested and stained using the T-reg detection Kit (Miltenyi Biotec) prior to flow cytometric analysis.

### Statistical analysis

All results were expressed as mean ± standard deviation (SD). Differences observed between groups were evaluated using the Wilcoxon/Mann-Whitney *U* test, a nonparametric test that does not assume normality of the measurements (it compares medians instead of means). Values of *p*<0.05 were considered statistically significant. All statistical analysis were performed with STATISTICA version 8.0.725.0 (StatSoft GmbH)

## Results

### Isolation of *E. multilocularis* larvae

**Figure 1:**
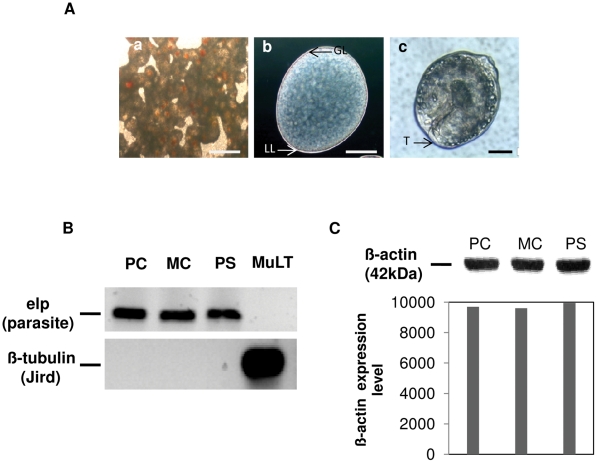
Isolation of *E. multilocularis* larval stages. (**A**) Morphology of *E. multilocularis* primary cell aggregates (a), metacestode vesicles (b), and protoscoleces (c), that were used for co-cultivation with host DC and other immune effector cells. Primary cell aggregates, representing the oncosphere-metacestode transition complex, were devoid of mature metacestode vesicles. Metacestode vesicles exhibited a cellular germinal layer (GL) and an acellular laminated layer (LL). Protoscoleces were covered by a syncytial tegument (T). White bar = 500 µm, Black bar = 50 µm. (B) Qualitative assessment of parasite material. To ensure that no host cell contamination is present in larval material prior to use, protoscoleces (PS), metacestode vesicles (MC), and primary cells (PC) had been cultivated for one week under axenic conditions. Chromosomal DNA was isolated and subjected to PCR for the *E. multilocularis*-specific gene *elp* (upper panel) and a jird-specific β-tubulin-gene (lower panel). As a control, *Meriones unguiculatus* liver tissue (MuLT) was used. (C) Quantification of larval material. To normalize larval material for cell numbers, the relative β-actin content was used. 2000 protoscoleces (PC), 4 metacestode vesicles (5 mm in diameter), and 1/6^th^ of the amount of primary cells that can be isolated from 40 ml MC vesicles (PC) were used to produce cell lysates. The lysates were subsequently separated on a 12.5% acrylamide gel and subjected to Western blot analysis using an anti-β-actin antibody (upper panel). β-actin band intensity was subsequently quantified using the ImageJ [39] analysis tool (lower panel).

The morphology of the three different *E. multilocularis* larval stages investigated in this study is depicted in Figure 1A. Primary cells were isolated from the GL of metacestode vesicles and seeded into culture dishes where they formed aggregates with central cavities within one week of cultivation. As previously outlined, these primary cell aggregates closely resemble the early developing metacestode both morphologically and physiologically, and routinely result in the production of fully mature metacestode vesicles after 3–4 weeks of cultivation [28]. Furthermore, primary cell aggregates express factors such as members of the EG95/W45 protein family (or host protective oncospheral antigens) that are specifically present in taeniid oncospheres and are known to play an important role in early parasite establishment [30]. Primary cell aggregates thus closely mimic the *E. multilocularis* larval stage at the onset of the oncosphere-metacestode metamorphosis [29], [30]. In all experiments, primary cell cultures were carefully checked for the absence of mature, LL-containing metacestode vesicles (Figure 1Aa). Mature metacestode vesicles had a size of approximately 5 mm (diameter) and were completely equipped with a LL surrounding a cellular GL (Figure 1Ab). Protoscoleces (50–100 µm in size) were covered by a tegument and were used in a non-activated, dormant state (i.e. no pre-activation with low pH and trypsin), as they typically occur within metacestode vesicles in the intermediate host (Figure 1Ac). After one week cultivation under axenic conditions [33], [35], the absence of contaminating, cellular host material in all parasite samples was confirmed by organism-specific PCR (Figure 1B). Parasite material of each of the three larval stages was subsequently normalized on the basis of β-actin content (Figure 1C; Figure S1). In all subsequent DC co-cultivation procedures, comparable amounts of parasite material were used with 1 Unit defined as 2000 protoscoleces, 4 metacestode vesicles of 5 mm of diameter, and 1/6^th^ of primary cells generated from 40 ml of metacestode vesicles after 1 week of *in vitro* culture (Figure 1C).

### E/S-products of *E. multilocularis* larvae induce apoptosis in DC

**Figure 2:**
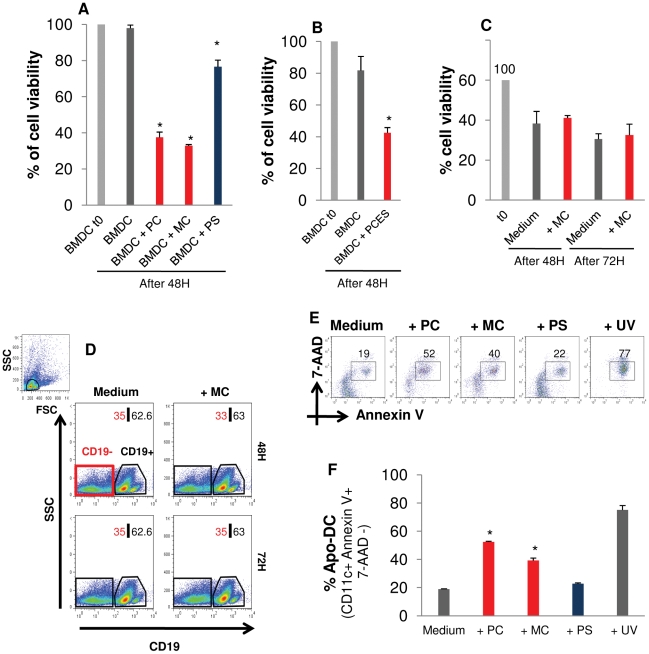
E/S-products of *E. multilocularis* larvae induce DC Death, but fail to kill splenocytes. (A) DC were exposed to comparable amounts of parasite material (1 Unit) from each of *E. multilocularis* larval stages (primary cells, PC; metacestode vesicles, MC; protoscoleces, PS), separated through a transwell system, or (B) to primary cell aggregate-conditioned medium (PCES) for 48 h. DC viability was then assessed using the Trypan Blue exclusion test. The number of surviving DC is expressed as a percentage of the initial number of DC seeded. As a control, the percentage of viable DC at the beginning of the experiment is given (t0). Results shown are the means+− SD of 4 independent experiments. * = p<0,05. (C) Splenocytes from female C57BL/6 mice were similarly exposed to metacestode vesicles (MC), separated through a transwell system, and viability was assessed using the Trypan Blue exclusion test after 48 h and 72 h. (D) Within the splenic lymphocytes, gated with respect to the forward and side scatter, CD19+ lymphocytes were stained and CD19− lymphocytes deduced by flow cytometry and the proportions of both cell populations (CD19− and CD19+) were monitored for changees upon exposure to MC. C and D are representative of two independent experiments. (E, F) E/S-products of *E. multilocularis* larvae induce DC death via apoptosis. DC were exposed to comparable amounts of *E. multilocularis* larvae (primary cell aggregates, PC; metacestode vesicles, MC; protoscoleces, PS), separated by a transwell system, for 24 h. DC were then analyzed by flow cytometry for the expression of Cd11c, AnnexinV and 7-AAD. The proportion of apoptotic DC (Cd11c+AnnexinV+7-AAD−) was then determined. UV-treatment of DC was used as positive control for apoptosis-inducing conditions. (E) Representative plots of the proportion of AnnexinV+ 7-AAD− cells, gated on CD11c+ cells. (F) Mean percentage +− SD of apoptotic DC (Apo-DC) of 4 independent experiments. * = p<0,05.

In a first set of experiments, the influence of *E. multilocularis* E/S-products on DC viability was tested. To this end, *E. multilocularis* larval material (1 Unit each for all three larval stages) was co-incubated for 48 h with DC, physically separated through a transwell system (1 µm pore size), and the number of viable DC was assessed by trypan blue exclusion. As shown in Figure 2A, co-incubation of DC with primary cells and metacestode vesicles led to greatly reduced viability (30–40% surviving cells compared to the control), whereas a still significant, but reduced killing effect was observed in the presence of protoscoleces (76,6+/−3,7% survival). To exclude the possibility that cell death in DC-parasite co-cultures merely resulted from starvation due to the presence of proliferating larvae, conditioned medium of a comparable amount of primary cells was tested on DC. To this end, supernatant from primary cell cultures (7 days old) was collected, sterile filtered, and added to fresh cultures of DC. As depicted in Figure 2B, even in the absence of proliferating larvae, conditioned medium similarly induced DC death.

In order to assess whether *Echinococcus* E/S-products do have general cytolytic effects that might account for the observed DC death, a hemolysis assay was carried out in which parasite material was co-incubated with human erythrocytes. However, as shown in Figure S2, no such effects were observed for any of the larval stages. We further tested whether *E. multilocularis* metacestode vesicles, which displayed the strongest killing effect on DC, could also affect other immune effector cells. Splenocytes from C57BL/6 mice were exposed to metacestode vesicles through a transwell system for 48 to 72 h and host cell viability was assessed by trypan blue exclusion. As shown in Figure 2C, in contrast to what we observed for BMDC, splenocyte viability was not affected by E/S-products of metacestode vesicles. We also specifically analyzed the CD19+ (B cells) and CD19− (primarily T cells) splenic lymphocytes for the effect of MCE/S on viability. As observed for the whole splenocyte population, the splenic B and T cell proportions were not altered upon 48–72 h of exposure to MCE/S (Figure 2D). Taken together, these data indicated that E/S-products of *E. multilocularis* larvae, particularly those that are released by primary cells and metacestode vesicles, induce murine DC death, but do not have general cytolytic properties and do not lead to killing in whole spleen cell preparations or alter the splenic B and T cell compartments *in vitro*.

To examine by which mechanism the *E. multilocularis* E/S-products induced DC death, Annexin-V/7-AAD dual staining was performed to differentiate necrotic (7AAD^+^) from apoptotic (Annexin-V^+^) cells. DC were separately exposed to comparable amounts of parasite material from each of the larval stages through transwells for 24 h, harvested and processed for staining. As shown in Figure 2E,F, following exposure of DC to E/S-products of primary cells or metacestode vesicles, 2-fold more DC underwent apoptosis than DC exposed to protoscolex E/S-products, which showed a similar rate of apoptosis as untreated DC. Taken together, these results indicated apoptosis as the primary mechanism by which E/S-products from primary cells and metacestode vesicles induce DC death.

### E/S-products of *E. multilocularis* larvae differentially affect DC maturation

**Figure 3:**
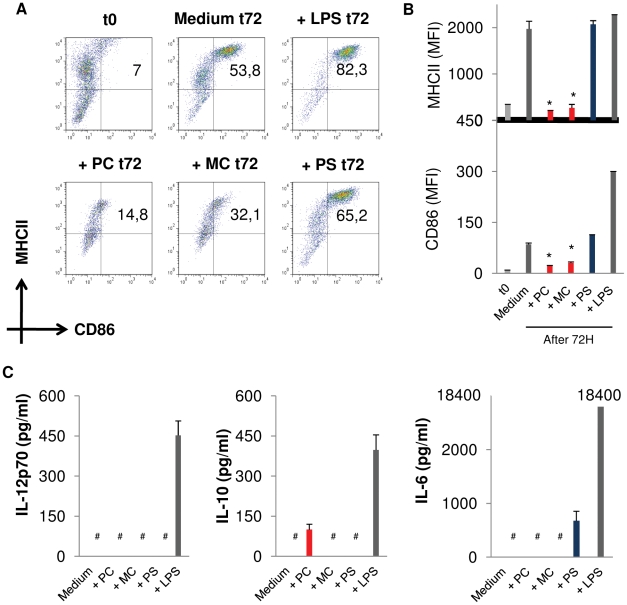
E/S-products of *E. multilocularis* differentially affect DC maturation and the DC cytokine profile. DC were exposed to *E. multilocularis* larvae (primary cell aggregates, PC; metacestode vesicles, MC; protoscoleces, PS), separated through a transwell system, for 72 h and their maturation was assessed by flow cytometry after staining for surface markers CD11c, MHCII and CD86. (A) Representative scatter plots showing the proportion of matured DC (upper right quadrant) with respect to MHCII and CD86, gated on CD11c cells. t0 indicates maturation at the beginning of the experiment. (B) Mean fluorescence intensities of MHCII and CD86 on DC after 72 h of treatment. (C) DC supernatants were collected and analyzed for the presence of IL-12p70, IL-10 and IL-6 by ELISA. Data shown are the mean +− SD of 4 independent experiments. # : not detectable by ELISA.

Having shown that E/S-products of *E. multilocularis* primary cells and metacestode vesicles induce apoptosis in part of the co-incubated DC, we subsequently analyzed the fate of the surviving DC concerning maturation and cytokine release. To this end, DC were first incubated for 72 h with each of the three different *E. multilocularis* larval stages, separated by transwells. Subsequently, DC maturation was assessed by measuring the expression of surface markers MHCII and CD86 by flow cytometry (Figure 3A,B). Interestingly, E/S-products from primary cells and metacestode vesicles significantly inhibited the spontaneous DC maturation rate as compared to untreated DC, which was not observed for E/S-products of protoscoleces (Figure 3A, B).

To investigate whether parasite larvae also alter DC cytokine release, DC were first exposed to each of the parasite stages through transwells for 24 h after which the parasite larvae were removed. Upon this brief exposure to the various larvae, the DC were harvested, counted and an equal number of surviving DC were re-seeded in fresh medium for an additional 48 h. Supernatant was then harvested and the level of secreted IL-12, IL-6 and IL-10 was assessed by ELISA. As expected, bacterial LPS, used here as a positive control, led to a strong induction of all three cytokines in DC (Figure 3C). On the other hand, none of the parasite E/S-products was able to trigger IL-12 release. However, substantial amounts of IL-10 were produced upon exposure of DC to E/S-products of primary cells, whereas those of protoscoleces induced the production of IL-6. Following challenge by metacestode E/S-products, neither IL-6 nor IL-10 production were induced in DC (Figure 3C). Taken together, these results indicated that E/S-products of primary cells and metacestode vesicles inhibited the ability of DC to spontaneously mature *in vitro*, and failed to promote (or blocked) the release of detectable amounts of the pro-inflammatory and Th1-associated cytokine, IL-12. Interestingly E/S-products of primary cells additionally acted on DC to induce IL-10 secretion, a feature which was not seen with E/S-products of the metacestode. In contrast, E/S-products of protoscoleces fostered DC maturation and induced IL-6 but not IL-12 secretion by DC.

### E/S-products of *E. multilocularis* larvae modulate DC response to subsequent LPS exposure

**Figure 4:**
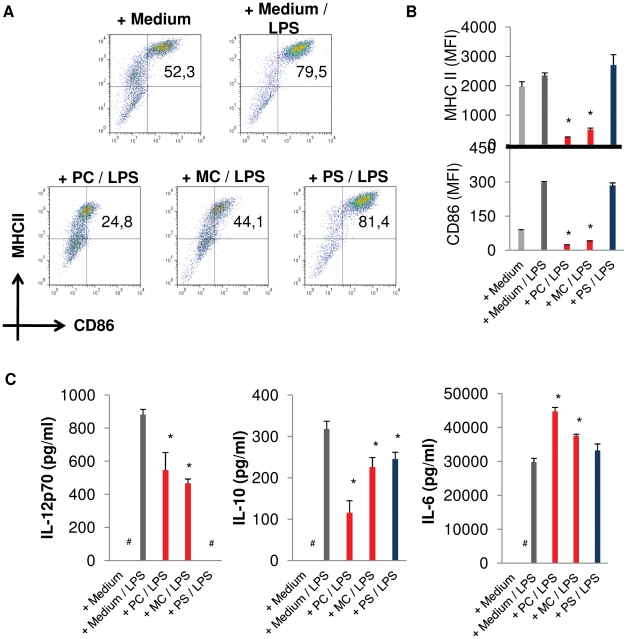
E/S-products of *E. multilocularis* differentially alter DC responsiveness to LPS. DC were first exposed to *E. multilocularis* larvae (primary cell aggregates, PC; metacestode vesicles, MC; protoscoleces, PS), separated through a transwell system, for 24 h. DC were then harvested, counted and seeded at equal numbers of living cells in medium containing LPS for 48 h, prior to staining and flow cytometric detection for surface markers CD11c, MHCII and CD86. (A) Representative scatter plots showing the proportion of matured DC (upper right quadrant) with respect to MHCII and CD86, gated on CD11c cells. (B) Mean fluorescence intensities of MHCII and CD86 on DC. (C) DC supernatants were collected and analyzed for the presence of IL-12p70, IL-10 and IL-6 by ELISA. Data shown are the mean +− SD of 4 independent experiments. * = p<0,05. # : not detectable by ELISA.

The consistent absence of detectable amounts of IL-12 in culture supernatant of DC after treatment with *E. multilocularis* larvae suggested that parasite E/S-products either failed to induce the secretion of this Th1-associated cytokine, or simply impaired its production by treated DC. Since there is increasing evidence that parasite survival within the intermediate host depends on the ability to deviate immune polarization away from a potential parasitocidic Th1 type [8], we examined whether exposure of DC to *E. multilocularis* E/S-products could have an influence on subsequent DC maturation by LPS, a stimulus usually associated with strong IL-12 release. To this end, DC were first incubated with each of the larval stages physically separated through transwells for 24 h. After this brief incubation time, the parasite larvae were removed and the DC were harvested, counted and an equal number of surviving DC were re-seeded in fresh medium containing 0.1 µg/ml of LPS for an additional 48 h. At the end of this second incubation period, DC were assessed for their level of maturation (MHCII and CD86 surface molecule expression) and DC supernatant was analyzed by ELISA for the presence of IL12p70, IL10 and IL6. As shown in Figure 4A/B, in contrast to the situation for protoscolex co-cultures, LPS did not induce maturation when DC had previously been cultivated in the presence of primary cells or metacestode vesicles. Furthermore, pre-exposure to E/S-products of all three larval stages significantly altered the cytokine profile of DC upon subsequent LPS stimulation, again leading to a strongly diminished release of the Th1 associated cytokine IL-12 (Figure 4C). Taken together, our results indicate that exposure of DC to E/S-products of primary cells inhibits maturation in response to LPS, diminishes the ability to produce IL-12 and IL-10, but induces IL-6 production. E/S-products of metacestode vesicles act similarly on DC, but have a reduced effect on LPS-induced IL-10 and IL-6 production. In contrast, E/S-products of protoscoleces do not inhibit the expression of MHCII and CD86 surface markers by LPS-stimulated DC, but strongly affect LPS-induced IL-12 production.

### E/S-products of *E. multilocularis* larvae differentially affect DC-based CD4+CD25+Foxp3+ T cell conversion *in vitro*

**Figure 5:**
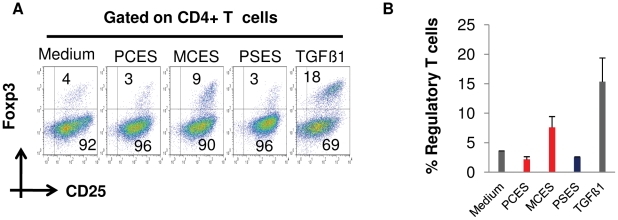
E/S-products of *E. multilocularis* metacestode vesicles induce CD4+CD25+Foxp3+ Tcell conversion *in vitro*. Freshly generated DC (Day 8) were co-cultured with naïve CD4+CD25− OT2 T cells at a DC∶T cell ratio of 1∶3 in medium supplemented with OVA peptide in the presence of conditioned medium of primary cell aggregates (PCES), metacestode vesicles (MCES) or protoscoleces (PSES). After 5 days of incubation, cells were harvested and stained for CD4, CD25 and Foxp3 prior to flow cytometry analysis. As a positive control for CD4+CD25+Foxp3+ T cell conversion, murine Transforming Growth Factor-β was used (TGF-β). (A) Representative plots depicting the proportion of CD4+CD25+Foxp3+ T cells (upper right quadrant) of pooled duplicates from 2 independent experiments, summarized in (B).

A recent report has suggested an implication of Foxp3+ regulatory T cells (T-reg) in *E. multilocularis* larval establishment and/or persistence within the intermediate host [15]. Furthermore, a key role of DC in inducing the *de novo* generation of Foxp3+ T-reg is well established [41]–[43]. Using an in vitro OVA peptide-based assay for the co-cultivation of DC and CD4+ T cells [44] we therefore tested whether additionally present E/S-products of *E. multilocularis* larvae could lead to an expansion of CD4+CD25+Foxp3+ T cells, commonly assumed to be T-reg [41]–[43]. To this end, co-cultures of freshly generated DC (day 8) and naïve CD4+CD25− T cells from OT2 αβ TCR-transgenic mice at a DC/T cell ratio of 1/3, supplemented with OVA peptide, were exposed to E/S-products of all three larval stages. Upon 5 days of incubation, the cells were harvested and stained for Foxp3+ T-reg-specific cell markers (CD4, CD25 or IL2Rα chain and Foxp3). Interestingly, in contrast to co-cultures of DC and T-cells with E/S-products of primary cells and protoscoleces, there was a significant expansion (2.5-fold) of the population of T-reg (CD4+CD25+Foxp3+) in co-cultures that included E/S-products of metacestode vesicles (Figure 5). These results indicated that at least the developmental stage that characterizes the chronic phase of AE, the metacestode, is able to induce *de novo* CD4+CD25+Foxp3+ T-reg conversion *in vitro* in the presence of DC.

## Discussion

As typical in the case of helminth infections, AE is a long-lasting and chronic disease that is most probably associated with parasite-induced, immunosuppressory mechanisms around the primary site of infection [8]. For a number of nematode and trematode systems, research during recent years has demonstrated a crucial role of T-reg in the respective immunosuppressive mechanisms and emphasized the importance of DC in the induction of helminth-associated Th2- and tolerogenic immune responses [11], [12]. Compared to nematode and trematode infections, immunomodulatory functions of DC in cestode infections have drawn significantly less attention, although this is clearly an emerging field since several studies concerning the influence of parasite products on DC maturation and cytokine secretion profiles have been conducted very recently. In two of these recent reports, Reyes *et al.* [45] and Terrazas *et al.* [46] investigated the effects of E/S-products of *Taenia crassiceps* cysticerci, representing the metacestode larval stage of this *Taenia* infection model, on the activation of murine DC. These authors observed impaired DC maturation in response to TLR dependent stimuli, particularly when DC of infection susceptible mouse strains were pre-incubated with parasite E/S-products [45]. In the case of *E. granulosus*, a species closely related to *E. multilocularis*, the effects of hydatid cyst fluid (HCF) and isolated antigen B (AgB), a major constituent of HCF, were tested and led to DC maturation as well as DC cytokine profiles that were indicative of Th2 immune responses [19], [20]. However, whether these interactions are of major relevance *in vivo* remains questionable since intact parasite tissue usually prevents direct contact between HCF and host immune effector cells, and the spectrum of metacestode E/S-products does not necessarily overlap with the spectrum of proteins present in HCF. Although it is generally assumed that AgB might leak out of intact metacestode vesicles or be released early during an infection from damaged metacestode material [47], we could not detect AgB in the E/S-products of *in vitro* cultivated *E. multilocularis* metacestode vesicles despite the fact that this component was well expressed in HCF [48]. Crude metacestode antigen preparations containing vesicle fluid, somatic parasite proteins and contaminating host components [16] as well as isolated vesicle fluid of *E. multilocularis* [20] were also already tested concerning their effects on DC and failed to induce maturation as did a purified mucin-type glycoprotein (Em2) that is usually expressed at the surface of LL-containing metacestode vesicles [49], [50]. Hence, although all these reports indicate that larval cestode parasite products can exert immunomodulatory effects on host DC, depending on the source and form of application, their precise nature and role during the course of an infection of the intermediate host remains elusive so far. What became clear through a recent *in vivo* investigation on experimentally infected mice, on the other hand, was that, at least in the chronic phase of experimental AE, peritoneal DC display a significant down-regulation of surface markers that are associated with DC maturation, and over-expressed TGF-β mRNA, which might lead to an induction of T-reg in this phase of the disease [15], [51]. A potential role of T-reg in human AE has also already been suggested [8] based on the fact that immune-suppressive TGF-β and IL-10, major cytokines that are released by T-reg [52], can be predominantly found in the immediate vicinity of actively proliferating parasite tissue.

In order to more closely mimic the situation at the site of infection, we utilized in this study a cultivation system by which actively secreted E/S-products of living parasite material can be tested on DC. Furthermore, in addition to parasite components that are produced during late stages of the infection (metacestode and eventually protoscoleces), we also included larval material that represents early stages of the infection, prior to the establishment of metacestode vesicles, in which *E. multilocularis* should be highly susceptible to host attacks due to the absence of a protective LL [7].

First, although co-incubation with protoscolex E/S-products clearly induced DC maturation, no activation could be observed upon co-incubation with E/S-products of primary cells and metacestode vesicles. The cells were not only affected in the expression of surface activation markers but also failed to secrete pro-inflammatory cytokines. This inhibitory effect was even apparent in the presence of strong stimuli of TLR signaling since DC pre-incubated with E/S-products of primary cells and metacestode vesicles did not mature in response to LPS, whereas pre-incubation with E/S-products of protoscoleces had no such effect. Furthermore, in a much more pronounced manner than protoscolex compounds, E/S-products of both primary cells and metacestode vesicles induced DC death mediated by apoptosis. To our knowledge, this is the first report on the induction of apoptosis in host DC in response to cestode larval material, which should have important implications concerning immuno-suppressive activities, particularly at the very early stage of the infection. On the one hand, the induction of apoptosis in DC should be beneficial to the parasite since it depletes immune effector cells around the early parasite lesions that are important to induce inflammatory immune responses. Moreover, it is well established that apoptosis, extrinsically triggered by infectious agents such as viruses, parasites, or bacteria, usually results in a bystander effect of induced immunosuppression [53]. In parasitic helminths, the induction of DC apoptosis has already been reported for microfilariae of the nematode *Brugia malayi* which, as also shown herein for *E. multilocularis*, strongly limited their capacity to produce pro-inflammatory IL-12, and prevented T cell activation and proliferation [54]. Previous *in vitro* studies further demonstrated that apoptotic DC are rapidly taken up by immature DC, which prevents subsequent maturation of immature DC in response to TLR stimuli [53]. It is, therefore, conceivable that the strongly diminished ability of DC that were pre-incubated with E/S-products of primary cells and the metacestode to LPS, as observed in our study, is indirectly mediated by the induction of apoptosis in a subset of immature DC, rather than by direct inhibition of DC maturation through parasite E/S-products. Since the uptake of apoptotic DC induces immature DC to secrete TGF-β, which induces differentiation of naïve T cells into Foxp3+ T-reg [53], E/S-products of the metacestode, and particularly of primary cells, could thus establish a strongly immunosuppressive environment around parasite lesions already at the beginning of an infection.

As in the case of E/S-products produced by *B. malayi* microfilariae [54], we can currently only speculate about the molecular nature of *Echinococcus* E/S factors that might induce DC apoptosis. Among the various host-derived compounds that can extrinsically trigger DC apoptosis are ligands of the tumor necrosis factor (TNF) superfamily as well as glucocorticoids [53] and *B. malayi* microfilariae have already been demonstrated to induce DC apoptosis by triggering TNFα-dependent signaling mechanisms [55]. Although no TNF-like ligand has been described so far in *Echinococcus* or any other flatworm, there has been a recent report on the presence of a TNFα-receptor like surface protein in *Schistosoma mansoni* which presumably interacts with host TNFα [56]. Our own preliminary analyses on the *E. multilocularis* genome, which is currently being sequenced [29], [30], revealed that a very similar receptor is also expressed by cestodes (data not shown). Bioinformatically, TNF-ligands are difficult to identify in raw sequence data, which might be the reason why so far no such molecule was identified in the *Schistosoma* or *Echinococcus* genomes. However, the presence of a respective receptor in these organisms implies that they might also express cognate ligands, which subsequently could bind to members of the TNF-receptor family on host cells (such as DC), thus triggering apoptosis. Apart from components of the TNF signaling machinery, it has recently also been demonstrated that cestode larvae (*T. crassiceps* cysticerci) are capable of producing steroid hormones [57]. Although in this system only the production of sex steroids has been tested, it is conceivable that they also produce glucocorticoids which might, either together with TNF-ligands or as an alternative, be involved in triggering host DC apoptosis. By utilization of the culture system established in this work, these alternatives can now be addressed.

A marked difference between DC that were incubated in the presence of E/S-products of metacestode vesicles and primary cells was that, in the latter case, the production of anti-inflammatory IL-10 was significantly induced. To our knowledge, an elevated expression of IL-10 coinciding with DC apoptosis has so far never been described, indicating that this effect was not provoked by the elevated induction of DC apoptosis through E/S-products of primary cells, when compared to those of the metacestode. Hence, we rather suggest that primary cells secrete a set of factors that differs from E/S-products of the metacestode and contains additional components that are able to induce the expression of IL-10 by non-activated DC. This hypothesis is supported by the differential influence of E/S-products from primary cells and metacestode vesicles that we have observed in T-reg conversion assays. Only E/S-products of metacestode vesicles, but not those of primary cells (or protoscoleces), were able to significantly increase the number of CD4+CD25+Foxp3+ regulatory T cells *in vitro*. Although we cannot presently tell whether the *in vitro* T-reg conversion was exclusively mediated by the modified DC, or whether there is also a direct influence of parasite E/S-products on CD4+ T cells, these data nevertheless clearly support an emerging picture that points at Foxp3+ T-reg cells as potential mediators of the fine tuning of the host immune system during metacestode establishment and growth within the intermediate host [15]. Furthermore, our data suggest that an expansion of Foxp3 expressing T cells during chronic (peritoneal) AE, as observed by Mejri et al. [15], might not simply be an intrinsic consequence of an ongoing immune response, but that the parasite actively induces Foxp3+ T-reg through its E/S-products.

The factor(s) and mechanism(s) involved in the modulation of DC maturation and function are currently subject to ongoing investigations. These include studies on the possibility that *Echinococcus* E/S-products might directly interact with TLR ligands rendering the latter less able to elicit a ‘normal’ response from DC, or may be acting as antagonists of TLR-ligand binding interactions [11]. Furthermore, parasite E/S-products may affect DC directly through interactions with non-TLR pattern recognitions receptors such as DC-SIGN, Dectin family members [11]. In previous studies on other helminth systems, secreted compounds such as filarial cystatins [58] were shown to induce the expression of IL-10 in antigen presenting cells, including non-activated DC. Interestingly, our own preliminary analyses of the *E. multilocularis* genome sequence indicate that related molecules are also encoded by the cestode, although further experimentation is clearly necessary concerning a possible secretion of these molecules by PC or whether they exert immunomodulatory activities comparable to those of filarial cystatins. Regarding T-reg conversion, the so far best characterized component that elicited similar *in vitro* effects as E/S-products from metacestode vesicles was a secreted compound of the nematode *Heligmosomoides polygirus* with TGF-β-like activities [59]. Due to the fact that TGF-β-signaling mechanisms have already evolved very early in animal evolution, TGF-β-like cytokines are expressed by a wide variety of free-living, but also parasitic invertebrates [60], [61]. Notably, at least one gene that encodes a structural homolog of mammalian TGF-β is also present on the genome of *E. multilocularis* [62], and the involvement of this component in the *in vitro* T-reg conversion process induced by metacestode E/S-products is currently investigated by us using the *in vitro* cultivation models established in this study.

In sharp contrast to co-incubation with primary cells and metacestode vesicles, DC exposed to E/S-products of protoscoleces were clearly activated, as assessed by up-regulation of surface activation markers (MHCII and CD86), secreted elevated levels of IL-6 (but no IL-10), and strongly impaired the ability of DC to produce IL-12 in response to TLR stimuli (LPS). This phenotype resembles that of DC that had been incubated in the presence of *E. granulosus* HCF and isolated AgB [19], [20]. However, in contrast to these investigations, DC incubated with protoscolex compounds in our study did not release elevated levels of IL-10, as reported by Rigano *et al.* [20], or IL-12, as reported by Kanan and Chain [19]. This is most probably due to the fact that the spectrum of E/S-products of protoscoleces does not fully overlap with the content of HCF since, for example, AgB is only weakly expressed by protoscoleces [30], [63]. In general, however, the phenotype of DC upon co-incubation with E/S-products of protoscoleces in our study is largely comparable to that of DC incubated with certain *Trypanosoma* antigens which have been closely associated with the induction of Th2-dominated immune responses [64]. Whether the Th2 immune response that is characteristic of the chronic stage of AE [8] is provoked (or supported) by direct contact between protoscoleces and DC within the intermediate host remains highly questionable, since this larval stage is only produced very late in the infection and direct contact between protoscoleces and host cells is usually prevented by the parasite's surface layers. Furthermore, Th2-dominated immune responses can also be observed in chronic AE under conditions in which no protoscoleces are produced [65]. However, since intestinal luminal infections by adult cestodes are associated with Th2 immune responses [66], the phenotype we observed in this study for DC exposed to E/S-products of protoscoleces could rather be associated with immunological processes that are relevant for an infection of the definitive host [66]. In any case, the marked differences between the responses of DC to E/S-products of early versus late developmental stages of *E. multilocularis* clearly demonstrates that an induction of tolerance in DC is not a general characteristic of *Echinococcus* material, but rather that the E/S repertoire of primary cells and metacestodes has specifically evolved to fulfill these purposes. Care should therefore be taken in the interpretation of results that have been obtained concerning the immune response during echinococcosis (intermediate host infection) by using co-incubation-systems of *Echinococcus* protoscoleces with host cells [21]–[27] or by employing the mouse model of peritoneal, protoscolex-induced secondary alveolar echinococcosis for short-term infections [67].

In conclusion, in this study we provide for the first time evidence for the induction of apoptosis in host DC through E/S-products of early infectious stages of *E. multilocularis*. We further show that primary cells, as representative of the oncosphere stage that undergoes metamorphosis towards the metacestode, are able to induce poorly responsive, IL-10 secreting DC *in vitro*. This effect is somewhat reduced at the chronic stage (metacestode), leading to poorly responsive, immature DC, but a Foxp3+-T-reg-inducing environment, and is no longer present in the protoscolex stage (table 1). Although our study concentrated on *in vitro* interactions between parasite larvae and DC, thus excluding the possible influence of other immune effectors or epithelial cells, the clear induction of poorly responsive, apoptotic and IL-10 secreting DC in response to primary cells suggests that a similar mechanism might also be operative in the tissue surrounding the early metamorphosing oncosphere. If so, this process might be important for an early establishment of the parasite during a phase of relatively high vulnerability to the host immune system, whereas in the chronic phase, after production of the LL, a slightly altered profile of E/S-products that mainly induces T-reg could support long-term persistence and infiltrative growth of the metacestode, as previously suggested [15]. The molecular nature of *Echinococcus* E/S-products that are responsible for these effects is currently being investigated by us using the available genome sequence information [29], [30], recently established methods for genetic manipulation of primary cells [68], and the cultivation settings established in this work.

**Table 1:** *In vitro* immunomodulation of DC by E/S-products of *E. multilocularis* larvae.

	Larval stages		
	Primary cells	Metacestode vesicles	Protoscoleces
Induction of apoptosis	**++**	**+**	**±**
Surface maturation markers(MHCII, CD86)	↓↓	↓	↑
Induction of IL-12p70	**−**	**−**	**−**
Induction of IL-10	**+**	**−**	**−**
Induction of IL-6	**−**	**−**	**+**
IL-12p70 production upon subsequent exposure to LPS	↓	↓	↓↓↓
*De novo* DC-based CD4+CD25+Foxp3+ T-reg conversion	−	**+**	−
Classification of resulting DC	DC killing + IL-10-producing DC	DC killing + Tolerogenic Foxp3+ T-reg-inducing DC	Th2-like inducing DC?

## Supporting Information

Figure S1 **Normalization of parasite material.** (A) Different quantities of host-cell free *E. multilocularis* larvae were used to generate cell lysates, which were subsequently analyzed by Western blot using an anti-β-actin antibody (upper panel). β-actin band intensity was subsequently quantified by ImageJ (lower panel). PC1, primary cells from 15 ml of metacestode vesicles after 1 week axenic cultivation; PC2, primary cells from 25 ml of metacestode vesicles after 1 week axenic cultivation; V1, 1 metacestode vesicle of 5 mm diameter after 2 months in vitro cultivation and one week axenic cultivation; V2, 2 metacestode vesicles of 5 mm diameter after 2 months in vitro cultivation and one week axenic cultivation; Pro1 and Pro2, 750 protoscoleces each after 1 week axenic cultivation. (B) Based on the values obtained in (A), the starting material of larvae was adjusted to the quantity of PC which could generate metacestode vesicles within 2–4 weeks (2PC here defined as 1 Unit of primary cells) and again analyzed by Western blot directed against β-actin. 2PC, 1/6^th^ of the amount of primary cells isolated from 40 ml metacestode culture; 2V, 4 metacestode vesicles of 5 mm diameter after 2 months in vitro cultivation and 1 week axenic cultivation; 2Pro, 2000 protoscoleces after 1 week axenic cultivation. (C) Western blot analysis of half of the amount of normalized parasite material determined in (B). (D) Assessment of the total amount of protein extracted from 1 Unit each of larval material.
(TIF)

Figure S2 **Assessment of the hemolytic activity of** ***E.multilocularis*** **E/S-products.** Human blood was collected from 2 healthy donors in heparinized tubes and, for each donor, an equal volume of red blood cell suspension was seeded in parasite culture medium. Red blood cells were exposed to E/S-products of comparable amounts (1 Unit) of primary cells (PCES), metacestode vesicles (MCES) and protoscoleces (PSES), and maintained in culture. Culture medium (Medium) was used as negative control whereas an equal volume of water was used as positive control (H2O). After 48 h, the plates were centrifuged and the supernatant collected for measurement of hemoglobin content as a marker of red blood cells lysis. The percentage of hemolysis is expressed as a ratio of sample absorbance over that of water (540 nm). Results shown are means +− SD from 2 healthy donors.
(TIF)
