# Supplementary figures and images for: Excretory/Secretory-Products of Echinococcus multilocularis Larvae Induce Apoptosis and Tolerogenic Properties in Dendritic Cells In Vitro
Source: PLoS Negl Trop Dis. 2012 Feb 21;6(2):e1516. doi: 10.1371/journal.pntd.0001516 (PMC3283565; doi:10.1371/journal.pntd.0001516)

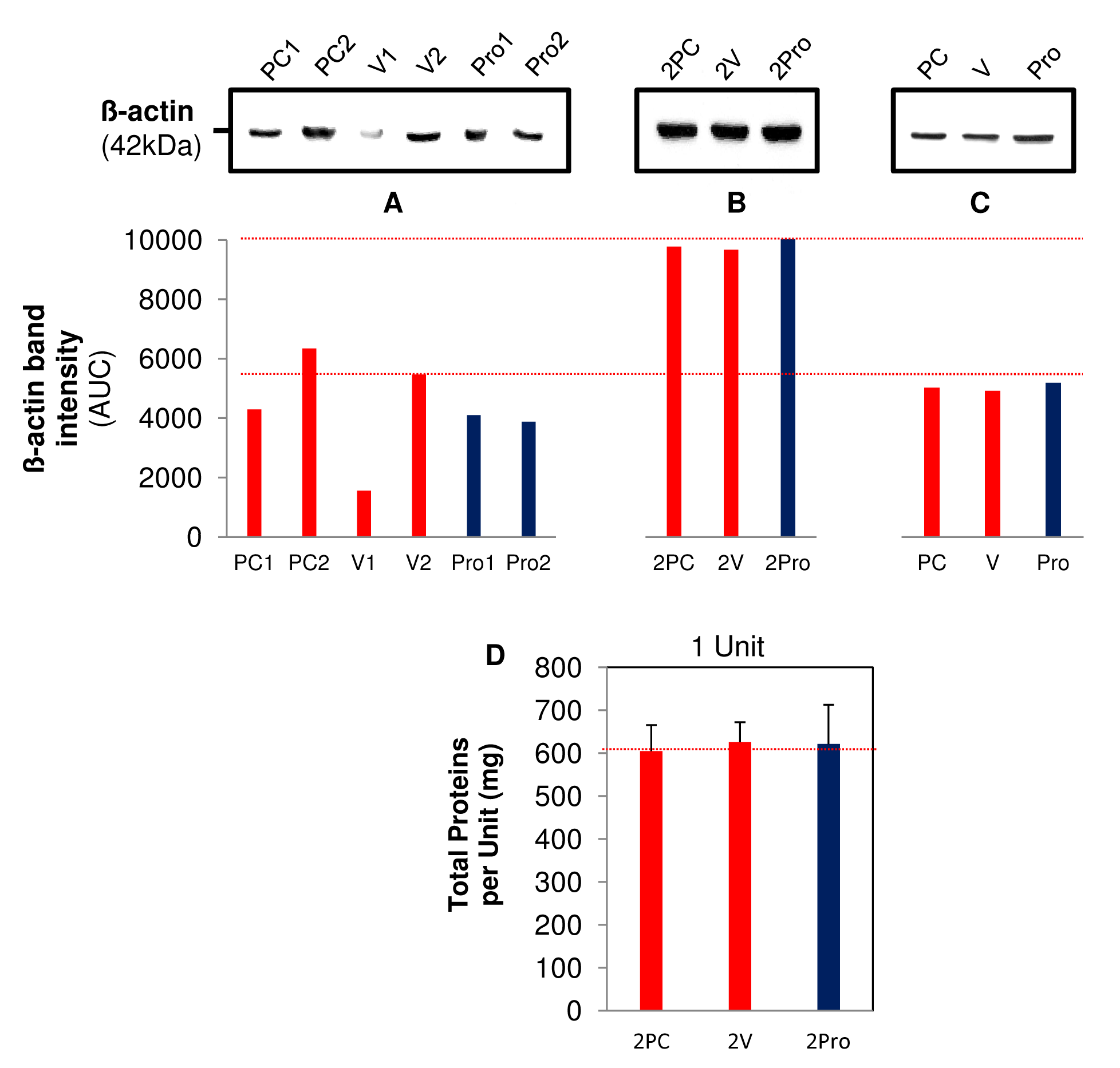

Supplement: Figure S1 — Normalization of parasite material. (A) Different quantities of host-cell free E. multilocularis larvae were used to generate cell lysates, which were subsequently analyzed by Western blot using an anti-β-actin antibody (upper panel). β-actin band intensity was subsequently quantified by ImageJ (lower panel). PC1, primary cells from 15 ml of metacestode vesicles after 1 week axenic cultivation; PC2, primary cells from 25 ml of metacestode vesicles after 1 week axenic cultivation; V1, 1 metacestode vesicle of 5 mm diameter after 2 months in vitro cultivation and one week axenic cultivation; V2, 2 metacestode vesicles of 5 mm diameter after 2 months in vitro cultivation and one week axenic cultivation; Pro1 and Pro2, 750 protoscoleces each after 1 week axenic cultivation. (B) Based on the values obtained in (A), the starting material of larvae was adjusted to the quantity of PC which could generate metacestode vesicles within 2–4 weeks (2PC here defined as 1 Unit of primary cells) and again analyzed by Western blot directed against β-actin. 2PC, 1/6th of the amount of primary cells isolated from 40 ml metacestode culture; 2V, 4 metacestode vesicles of 5 mm diameter after 2 months in vitro cultivation and 1 week axenic cultivation; 2Pro, 2000 protoscoleces after 1 week axenic cultivation. (C) Western blot analysis of half of the amount of normalized parasite material determined in (B). (D) Assessment of the total amount of protein extracted from 1 Unit each of larval material. (TIF) [file pntd.0001516.s001.tif]

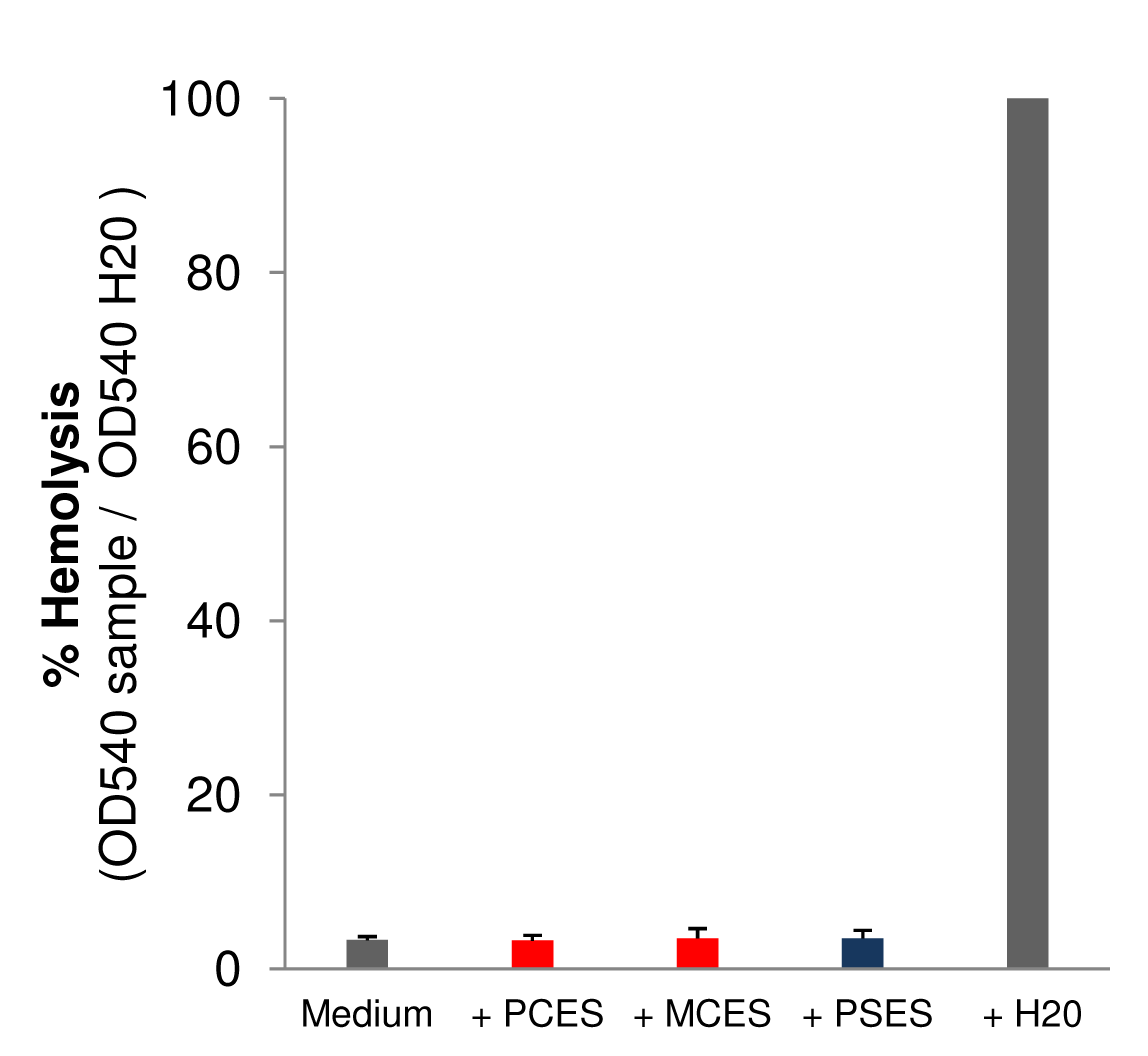

Supplement: Figure S2 — Assessment of the hemolytic activity of E.multilocularis E/S-products. Human blood was collected from 2 healthy donors in heparinized tubes and, for each donor, an equal volume of red blood cell suspension was seeded in parasite culture medium. Red blood cells were exposed to E/S-products of comparable amounts (1 Unit) of primary cells (PCES), metacestode vesicles (MCES) and protoscoleces (PSES), and maintained in culture. Culture medium (Medium) was used as negative control whereas an equal volume of water was used as positive control (H2O). After 48 h, the plates were centrifuged and the supernatant collected for measurement of hemoglobin content as a marker of red blood cells lysis. The percentage of hemolysis is expressed as a ratio of sample absorbance over that of water (540 nm). Results shown are means +− SD from 2 healthy donors. (TIF) [file pntd.0001516.s002.tif]
